# Discovery of metabolite biomarkers: flux analysis and reaction-reaction network approach

**DOI:** 10.1186/1752-0509-7-S2-S13

**Published:** 2013-12-17

**Authors:** Limin Li, Hao Jiang, Yushan Qiu, Wai-Ki Ching, Vassilios S Vassiliadis

**Affiliations:** 1 Institute of Information and System Science, Xi'an Jiaotong University, Xi'an, China; 2 Department of Mathematics, School of Information, Renmin University of China, Beijing, China; 3 Advanced Modeling and Applied Computing Laboratory, Department of Mathematics, The University of Hong Kong, Pokfulam Road, Hong Kong; 4 Department of Chemical Engineering and Biotechnology, University of Cambridge, Pembroke Street, Cambridge, UK

## Abstract

**Background:**

Metabolism is a vital cellular process, and its malfunction can be a major contributor to many human diseases. Metabolites can serve as a metabolic disease biomarker. An detection of such biomarkers plays a significant role in the study of biochemical reaction and signaling networks. Early research mainly focused on the analysis of the metabolic networks. The issue of integrating metabolite networks with other available biological data to reveal the mechanics of disease-metabolite associations is an important and interesting challenge.

**Results:**

In this article, we propose two new approaches for the identification of metabolic biomarkers with the incorporation of disease specific gene expression data and the genome-scale human metabolic network. The first approach is to compare the flux interval between the normal and disease sample so as to identify reaction biomarkers. The second one is based on the Reaction-Reaction Network (RRN) to reveal the significant reactions. These two approaches utilize reaction flux obtained by a Linear Programming (LP) based method that can contribute to the discovery of potential novel biomarkers.

**Conclusions:**

Biomarker identification is an important issue in studying biochemical reactions and signaling networks. Two efficient and effective computational methods are proposed for the identification of biomarkers in this article. Furthermore, the biomarkers found by our proposed methods are shown to be significant determinants for diabetes.

## Background

Deficiency in essential metabolites can directly cause metabolic diseases. Metabolic diseases profiling is promising in uncovering the mechanism of disease-metabolite associations. Existing research mainly emphasized on the analysis of metabolic networks [1-3]. Models in investigation of large-scale metabolic networks outperform other quantitative approaches [4,5]. The widespread appearance of gene expression data gives a clue for the integration of metabolite network data to reveal significant biomarkers.

Flux Balance Analysis (FBA) [6] is a constraint-based and traditional approach for predicting flux distribution. It has been employed in [7] to identify a number of important metabolic reactions. Drug targets are adopted to reduce abnormal metabolites through formulating an optimal combinatoric problem on metabolic networks [8,9]. In [10], drug target prediction can be formulated as an integer linear programming model. A quantitative method based on two-stage FBA has been proposed in [11] for drug target identification. In [12], by profiling human metabolic reactions, a drug-reaction network was established for predicting enzyme targets. Here we develop a computational approach to identify metabolic biomarkers using human metabolic reactions incorporating disease-specific gene-expression data [13]. Metabolic biomarkers are metabolites demonstrating consistent variation in concentration in disease state; they can be very useful for a diagnostic purpose, see for instance [14]. As an efficient diagnostic tool and a safe evaluator for drug candidates, metabolomics will play an important role.

Furthermore, we also construct a RRN which provides a platform for ranking the significance of reactions by using the PageRank algorithm. The reaction network can be constructed in such a way that the nodes represent reactions and an edge is placed whenever the reactions share the common metabolite. Note that one reaction can have thousands of edges with the related reactions which makes the network extremely complicated. This issue can be addressed by identifying densely connected subgraphs. The clustering toolbox is therefore employed to find the subnetworks. The graph clustering is based on the assumption that a group of functionally related nodes are likely to highly interact with each other while being more separate from the rest of the network [15]. In [16], the challenge of the clustering network graphs was presented. In particular, the results of most methods are highly sensitive to their parameters and the predicted clusters can vary from one method to another. Here we focus on analyzing the cluster with the largest number of entities. The selected metabolic reactions provide a platform for us to draw the RRN which depicts the interactions of the reactions. For the ease of interpretation and visualization of the networks, we apply Cytoscape to construct the network. PageRank approach is a promising method to evaluate the importance of a webpage. We then integrate the PageRank algorithm and the FBA method to evaluate the significance of the reactions. We also propose simple statistical criteria to select significant reactions which enable us to identify the corresponding metabolites.

Diabetes Mellitus is a group of metabolic diseases that are amongst the major human malnutrition diseases. Risk assessment is one of the possible ways to prevent the disease. Metabolic profiling, an unbiased technique, can potentially trigger the identification of high-risk candidates and therefore it can reduce the related costs [17].

We then integrate the human metabolic network with disease-specific gene expression data to analyze the flux profiles within the network. In the following section, we introduce two methods for metabolite biomarkers discovery. The validity of the two approaches will be further discussed. The identified metabolite biomarkers may have potential applications for disease diagnosis.

## Materials and methods

### Materials

The genome-scale human metabolic network reconstructed by Duarte et al. [18] consists of 3742 reactions, 2766 metabolites and 1905 genes. Three types of information have been used to describe a metabolic network. One of them is stoichiometry, which is used to depict the quantitative associations among reactants and products in all the involved reactions. Another part consists of enzymes corresponding to each reaction in the network. The last part is the flux capacity of each reaction. We employ Human Recon 1, one of the two independently developed human metabolic networks [18,19] in our study. The data is available at the BiGG database (http://bigg.ucsd.edu/). One can retrieve the reactions and the involved genes using MATLAB. And the RRN can be implemented in the Cytoscape software package which is available at (http://www.cytoscape.org).

### Methodology

We introduce two novel methods for integrating gene expression data and the human metabolic network in biomarker discovery.

### Flux profile comparison (FPC) method [13]

There are several major steps that we have to conduct before the construction of LP model, and eventually allow us for the detection of biomarkers.

1. **Expression levels in reactions**

2. **The LP model**

3. **Flux profiles in disease/normal samples**

4. **Identification of significant reactions**

5. **Significant metabolite discovery**

Comparing to the well known model using the human metabolic network to predict metabolic biomarkers of human inborn errors of metabolism [14], our model takes more realistic constraints into consideration. Firstly, the genome-scale human metabolic network we utilize here consists of 1905 boundary metabolites and 3742 reactions in total. Secondly, we integrate gene expression data in both normal and disease state to mark highly and lowly expressed reactions. Without forcing the reactions to be active in the normal state or inactive in the disease state, we adopt a probability measure for the reaction to be active or inactive instead. We use two pairs of gene expression data in both healthy and disease status and consider the overlap of the discovered metabolic biomarkers. Regarding the solutions of the LP problems, we use a large-scale optimization method which is based on Linear Interior Point SOLver (LIPSOL) [20] in MATLAB on a Windows Vista machine. These characteristics of our approach contribute to the discovery of metabolic biomarkers in a more significant way.

### RRN construction method

In this section, we propose the second novel approach to identify the metabolite biomarkers based on RRN.

• Clustering toolbox for identifying the subnetworks.

**Figure 1:**
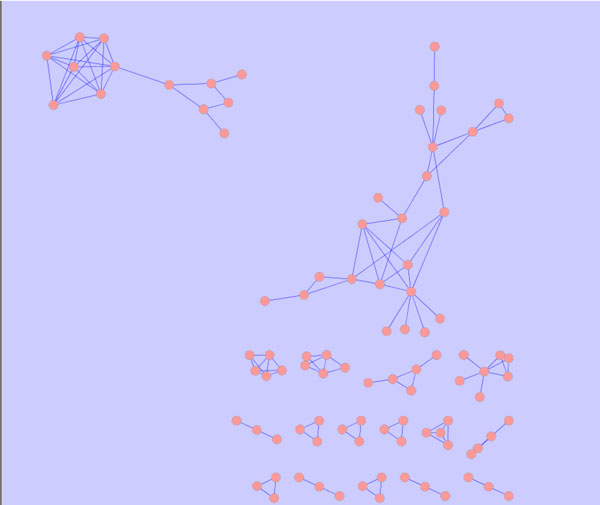
**Reaction network**.

The availability of the human metabolic network from Duarte et al. [18] enables us to retrieve the reactions and the genes. It includes 3742 reactions with 2766 metabolites and 1905 genes, which suggests a potential way to build a network if the reactions share the common metabolites. The nodes of the network represent the reactions, while the reactions are linked if they have the same metabolite. Some reactions have thousands of edges based on the foundation of constructing the network. Therefore the RRN can be extremely complex which makes it difficult to draw the network by using Cytoscape. We address this problem by using a clustering toolbox to identify the subnetworks which have similar properties. Clustering analysis [21] aims to classify a set of observations into two or more mutually exclusive unknown groups based on combinations of variables. Thus, cluster analysis is usually adopted in the context of unsupervised classification [22]. It can be applied to a wide range of biological study cases, such as microarray, sequence and phylogenetic analysis [23]. The purpose of clustering is to group different objects together by observing common properties of elements in a system. In a biological network, this can help identify similar biological entities, like proteins that are homologous in different organisms or that belong to the same complex and genes that are co-expressed [24,25]. Among all the clustering algorithms, the *k*-means algorithm [26] aims to partition *n *observations into *k *clusters in which each observation belongs to the cluster with the nearest mean. The *k*-means method and its modifications are widely used for gene expression data analysis [27]. Here we utilize this method to classify the reactions into several groups. Reactions in the same cluster have similar behavior. We focus on analyzing the cluster with the largest number of elements and we choose *k *to be 50. With different *k *the classification is consequently different. It is interesting to note that the elements in the largest cluster are quite similar. Thus parameter *k *does not have much influence on the results of the cluster. We finally choose the largest cluster with 134 reactions for our analysis. And the network is shown in Figure 1.

• The PageRank algorithm for evaluating the reactions in the RRN.

**Figure 2:**
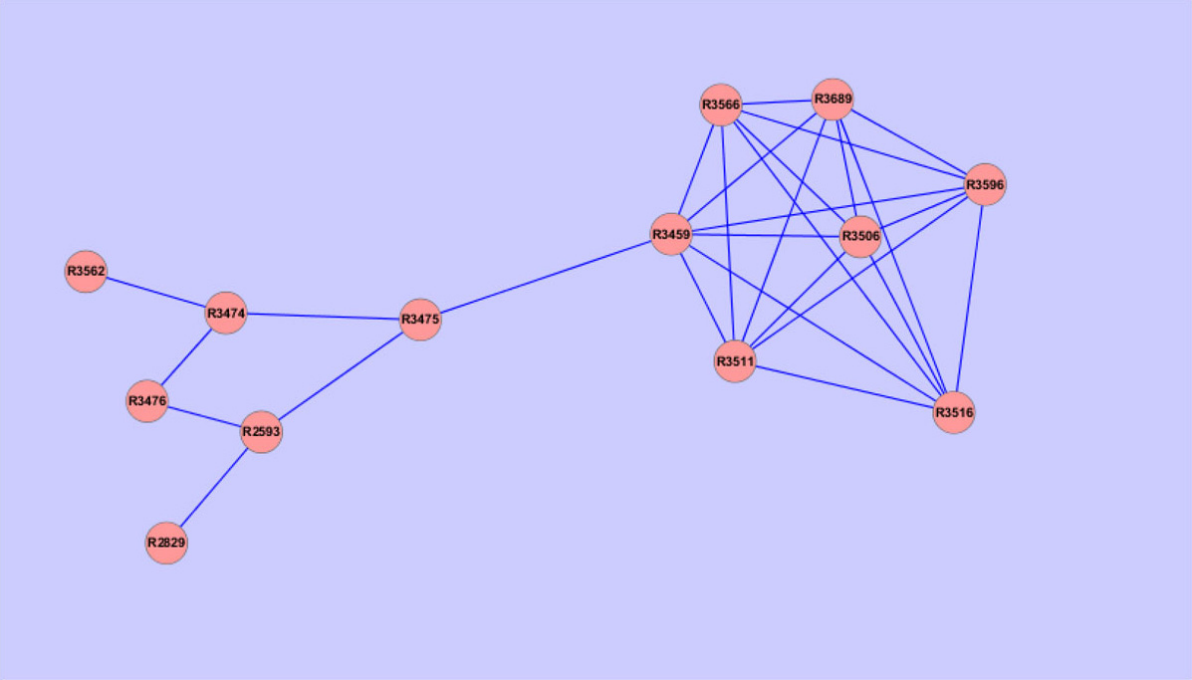
**The left upper corner of figure 1**.

**Figure 3:**
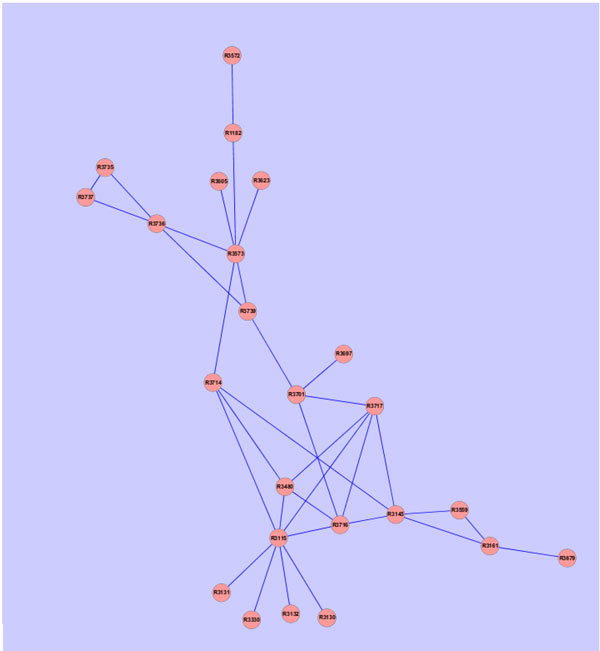
**The right upper corner of figure 1**.

With the obtained reaction network, one can evaluate the significance of each network. The page rank of a webpage is a number for representing the relative importance of the webpage based on the number of inbound and outbound links. Inbound links are links from outside pointing to a webpage. Outbound links are links from a webpage to any other webpages [28]. The page rank of a webpage can be obtained from the following formula:

$$P_{i}=\left(1-d\right)+d\underset{j\in M\left(i\right)}{\sum }\frac{P_{j}}{L\left(j\right)}$$

where *P_i _*is the page rank of the webpage *i*, *M *(*i*) is the set of the webpages linked to webpage *i*, *L*(*j*) is the number of outbound links of webpage *j*, and *d * is a residual probability which is usually set to be 0.85 [28]. Here we remark that the numerical results are similar in our experiments for other *d ≥ *0.85. The values of the PageRank of the webpages are the entries of the dominant eigenvector of the modified adjacency matrix. We denote the eigenvector

$$r={\left[P_{1},P_{2},\;\cdots \;,P_{N}\right]}^{T}$$

where *N* is the total number of pages and **r **is the solution of the recursive formula:

$$r=\left[\begin{array}{c} 1-d\\ 1-d\\ \;\;\;\vdots \\ 1-d \end{array}\right]+d\left[\begin{array}{cccc} \ell \left(1,1\right) & \ell \left(1,2\right) & \cdots \; & \ell \left(1,N\right)\\ \ell \left(2,1\right) & \ddots & & \vdots \\ \vdots & & & \vdots \\ \ell \left(N,1\right) & \ell \left(N,2\right) & \cdots \; & \ell \left(N,N\right) \end{array}\right]\;r$$

and the adjacency matrix *ℓ*(*i, j*) is 0 if webpage *i *does not link to webpage *j*, and we have the normalization condition that, for each *j*

$$\overset{N}{\underset{i=1}{\sum }}\ell \left(i,j\right)=1$$

i.e., the sum of each column is 1. The value of the ranking indicates an importance of a particular page. Inspired by [29], one can apply the PageRank algorithm to rank the importance of the reaction in the RRN. Here we apply this approach on two subgraphs which are exactly the subsets of Figure 1. The subgraphs are described in Figures 2 and 3. Figure 2 is the left upper corner of Figure 1 and Figure 3 is the right upper side of Figure 1. Then we can evaluate the nodes (reactions) in these two subnetworks. Furthermore, each reaction also has the flux value which represents the significance. Here we propose a simple approach to integrate flux value and rank value to yield a final significant score for each reaction. Let **z **be the vector containing the final values used for ranking the reactions. We define

$$z_{k}=p_{k}\times v_{k}$$

where **p **is the ranking result obtained from the PageRank algorithm, **v **is the flux vector for all the reactions generating from the flux analysis, and *k *represents the *k*th entry (reaction) of the vector. We then select several reactions with the comparatively high value from the RRN using this criteria.

## Results and discussions

In this section, we discuss some of our findings by our proposed two approaches: FPC and RRN. For FPC, we have filtered out 5 reactions and all the participating genes in these reactions [13]. In terms of genes, we have identified 11 significant genes involved in diabetes, "ALDH" and its variants(9 in total), "HSD3B2" and "KHK". In [30], "ALDH" activity has been experimentally shown to be related to the increasing risk of large vessel disease in diabetes. Direct intra-pancreatic delivery of ALDH activity could be a potential feasible strategy for diabetes [31]. Furthermore, researchers have found that, ALDH2.487Lys allele was related to the decreasing prevalence odds in type II diabetes in a clinical study on diabetes [32]. "HSD3B2" is discovered highly expressed with regulation of FXR (farnesoid × receptor) while FXR agonists are an appearing therapeutic treatment for diabetes [33]. The value of "KHK" as a pharmacological target needs further verification [34], but it can be a possible biomarker in diabetes treatment.

Considering the metabolites related diabetes, "ac[e]" acting as an inhibitor, is very helpful for patients in clinical trials, see for example [35]. Both "nadph" and "nadp" are valuable metabolites in l-xylulose (l-xylulose is obtained by "nadph" and "nad" reduction with "d-xylulose" [36]) which is intensively used in diabetes diagnosis. While reaction 1951 is glycolaldehyde dehydrogenase, glycolaldehyde has been shown to play a significant role in diabetic cardiomyopathy [37]. And "pi" is a critical component in the disturbance of diabetes [38].

Furthermore, we analyze the significant reactions selected by integrating the flux analysis and the PageRank algorithm based on the subnetworks of the RRN. We filter out five reactions which are reported in Table 1. All the participating genes in these five reactions are listed in Table 2.

**Table 1:** Significant reactions selected from RRN

Index	Reaction Equation
3115	"[x]:h2o+prpncoa→3hpcoa"

3161	"[c]:pi+uri ⇆ r1p+ura"

3573	"[c]:h+nadh+q10→nad+q10h2'

3474	"[m]:fad+succ ⇆ fadh2+fum"

3511	"[m]:coa+tetpent6crn→crn+tetpent6coa"

**Table 2:** Significant genes for diabetes in RRN

Reactions Index	Genes
3115	"EHHADH"

3161	"UPP1" "UPP2"

3573	"TXNRD1"

3474	"SDHD" "SDHC" "SDHB" "SD-HA"

3511	"CPT2"

We remark that the symbol "⇆" means the reaction is reversible and "*→*" means the reaction is irreversible. The number inside the parentheses (.) is the quantity of the metabolite. For example, in the Reaction 3511 of Table 1, we need "coa[m]:tetpent6crn[m] = 1:1" to produce "crn[m]:tetpent6coa[m] = 1:1". In the associated genes of Table 2, "CPT2" and "SDHD" etc are the gene symbols.

Considering the genes involved in the reactions, we have identified 9 important genes participating in Diabetes in Table 2. In [39], experiments have shown that gene "EHHADH" is involved in mitochondrial fatty acid *β*-oxidation and the variants in "EHHADH" are associated with type 2 Diabetes. In [40], it is demonstrated that obese diabetes impairs the rhythmic expression of various genes, including "UPP2". It has been shown that Diabetes appears to be associated with increased levels of oxidative stress in [41]. And gene "TXNRD1" exhibited increases in oxidative stress. "SDHB", "SDHA", "SDHC" and "SDHD" are four protein subunits forming succinate dehydrogenase. The role of "SDH" needs further investigation, but it is suspected that malfunction of the SDH complex can cause a hypoxic response in the cell that leads to tumor formation. Pharmacological inhibition of the CPT system by the glycidic acid derivative etomoxir, an irreversible and nonisoform-specific active site inhibitor of CPTs, has been demonstrated to reduce fasting blood glucose in an animal model of type 2 diabetes mellitus [42].

From the perspective of the metabolites related to the disease, reaction 3115 is an active reaction involved in fatty acid cholesterol metabolism during prostate cancer progression [43]. Here "pi" is a determining factor in regulation of metabolism in diabetes [38] and "nadh" involves in lactate formation (3-4-hydroxyphenyl lactate formation) [43]. Both "fad" and "fadh2" play an important role in fatty acids metabolism [44]. The role of malonyl CoA as a key glucose-derived metabolite, an allosteric inhibitor of fatty acid oxidation, has attracted many attentions. Recent studies have investigated the effects of manipulating this metabolite in various tissues [45]. For the issue of diabetes, a study in [46] demonstrated that high levels of malonyl CoA and reduced fat oxidation enhance glucose disposal in primary human skeletal myocytes.

One can see that these two approaches are extremely different, but some of the metabolite biomarkers are the same. It has been shown that "nadh" and "pi" are the important factors for detecting diabetes. We can conclude that both our proposed methods are effective for identifying the metabolite biomarkers for diseases.

## Conclusions

In this paper, we first develop a computational method to identify significant genes and metabolites for metabolic diseases. LP based strategy is then utilized to obtain flux profiles in disease/normal samples. Gene expression data in two pairs of samples at disease/normal states contributes to discovering genes and metabolites that can be potential biomarkers. We then further present a second novel approach to identify the significant metabolites for the metabolic disease. We also employ the constraint-based flux distribution to analyze the metabolic network. The clustering method makes it possible to identify the subgraphs with the common properties, which is a key step to construct the RRN with Cytoscape. To evaluate the reaction in the network, we propose the PageRank algorithm to evaluate the node. We integrate the flux value and the rank result to select the significant reactions, from which the related metabolites biomarkers can be identified. The integration of genome-scale human metabolic network data with gene expression levels offers a new way for systematically identifying potential biomarkers.

## Competing interests

The authors declare that they have no competing interests.

## Authors' contributions

LL and HJ came up with the idea. WKC, LL, HJ and VSV designed the research. HJ and YQ performed the research and analyzed the results. WKC, HJ, LL, YQ and VSV wrote the paper. All authors read and approved the final manuscript.

## Declarations

The publication of this article has been funded by the GRF Grant, HKU CERG Grants, Hung Hing Ying Physical Research Grant and National Natural Science Foundation of China Grant Nos. 10971075 and S201201009985.

This article has been published as part of *BMC Systems Biology *Volume 7 Supplement 2, 2013: Selected articles from The 6^th ^International Conference of Computational Biology. The full contents of the supplement are available online at http://www.biomedcentral.com/bmcsystbiol/supplements/7/S2.
